# Nationwide Patterns in Mortality and Demographic Variations in Appendiceal Disease Complicated by Septicemia in the United States, 1999 to 2020

**DOI:** 10.7759/cureus.110052

**Published:** 2026-06-01

**Authors:** Laya Kairam, Amaan Alvi, Rhutuja Khokale, Vidya Spandana Vellanki, Vennela Talla, Aadesh Singh Parmar

**Affiliations:** 1 Internal Medicine, RVM Institute of Medical Sciences and Research Centre, Mulugu, IND; 2 Internal Medicine, King George's Medical University, Lucknow, Lucknow, IND; 3 Internal Medicine, School of Medicine, Dr. D. Y. Patil University, Mumbai, IND; 4 Internal Medicine, Navodaya Medical College, Raichur, IND; 5 General Medicine, Vydehi Institute of Medical Sciences and Research Centre, Bangalore, IND; 6 General Internal Medicine, Hinduhridaysamrat Balasaheb Thackeray (HBT) Clinic, Mumbai, IND

**Keywords:** age adjusted mortality rate, cdc mcd, diseases of appendix, retrospective study, septicemia

## Abstract

Introduction

Mortality from appendiceal disease in high-income settings is uncommon but not negligible, and septicemia is a recognized pathway to fatal outcome. The population-level burden of appendiceal disease deaths complicated by septicemia, along with its demographic distribution and temporal trends, has not been well characterized at the national level in the United States.

Aims

To quantify age-adjusted mortality from appendiceal disease with septicemia as a contributing cause among U.S. adults aged ≥25 years over 1999 to 2020, identify demographic subgroups with the highest age-adjusted mortality burden, and characterize temporal trends in age-adjusted mortality across the study period.

Methodology

We conducted a retrospective, population-based analysis using the CDC WONDER Multiple Cause of Death database. Cases were adults aged ≥25 years for whom appendiceal disease (International Classification of Diseases, Tenth Revision (ICD-10) K35-K38) was assigned as the underlying cause of death and septicemia (A41.0-A41.9) appeared among the multiple causes of death. Crude and age-adjusted mortality rates (AAMRs) per 1,000,000 population were calculated with 95% confidence intervals, age-standardized to the 2000 U.S. standard population. Temporal trends were analyzed using joinpoint regression to estimate annual percent changes (APCs).

Results

A total of 3,923 deaths met the inclusion criteria, representing 45.9% of all 8,549 appendiceal disease deaths in this population over the same period. The overall AAMR was 0.8 per million (95% CI 0.8-0.9). Age-adjusted mortality was higher in men than in women (1.1 vs 0.6 per million). Although White individuals accounted for the largest absolute number of deaths (n = 3,270; 83.4%), the highest AAMRs were observed among American Indian or Alaska Native (1.1 per million) and Black or African American (1.0 per million) populations, indicating that the per-population burden is disproportionately borne by these groups. Age-adjusted rates were essentially flat across urbanization categories, and 84.3% of deaths occurred in inpatient settings. Joinpoint analysis demonstrated a significant decline in AAMR between 2002 and 2016 (APC −2.55%; p < 0.05), followed by an increase between 2016 and 2020 (APC +5.79%; p < 0.05) that overlaps with the COVID-19 pandemic period and warrants cautious interpretation. Trend analysis was not feasible for racial groups other than White owing to suppression of cells with counts below 10.

Conclusions

Septicemia is implicated in nearly half of all fatal appendiceal disease in U.S. adults and is therefore a frequent contributing pathway in fatal appendiceal disease. Age-adjusted mortality declined significantly through 2016 but rose thereafter, with the most recent segment coinciding with pandemic-era disruptions to surgical and emergency care. The disproportionate age-adjusted burden among American Indian or Alaska Native, Black or African American, and male populations supports continued surveillance and further investigation into the clinical and structural factors that drive these inequities.

## Introduction

Appendicitis is among the most prevalent causes of acute abdominal pain and remains one of the most frequent indications for emergency abdominal surgery [1]. Surgical management dominates clinical practice across age groups; in a large cohort of 336,880 patients, 94.2% of elderly and 97.3% of middle-aged patients underwent operative intervention, yet mortality remained substantially higher in older adults, with a 22% greater death rate than in middle-aged patients [2]. Appendicitis incidence appears to be rising in industrialized regions, and males carry a higher case-fatality risk than females. These observations indicate that despite the procedural maturity of appendectomy and the wide availability of supportive care in high-income countries, fatal outcomes continue to occur and are unevenly distributed across demographic groups.

When appendicitis progresses to a fatal outcome, septicemia is frequently involved as a major contributing complication. Sepsis is defined as life-threatening organ dysfunction resulting from a dysregulated host response to infection, and septic shock represents its most severe form, carrying markedly higher mortality than sepsis without shock [3]. Post-operative sepsis is a recognized complication of appendectomy, with 311 of 72,538 patients (0.43%) in one large analysis developing the complication and 17 (5.47%) dying within 30 days; African American race, severe obesity, acute renal failure or dialysis dependence, disseminated malignancy, and open surgical approach have all been associated with increased risk [4]. Delays in diagnosis or treatment increase the likelihood of perforation, abscess formation, and systemic infection [5,6]. Comorbidities, virulence of causative organisms, and surgical delays are important modifiers of progression from localized appendiceal disease to systemic infection.

Despite evidence linking appendicitis, septicemia, and mortality at the clinical level, population-level data quantifying the burden of fatal appendiceal disease with septicemia as a contributing cause remain limited, particularly with respect to long-term temporal trends and demographic differences in age-adjusted mortality. Most existing evidence is derived from hospital-based cohorts, which capture only patients reaching surgical care and therefore cannot characterize the national mortality burden or its distribution across population subgroups. Accordingly, the present study uses the Centers for Disease Control and Prevention’s Wide-ranging Online Data for Epidemiologic Research Multiple Cause of Death (CDC WONDER MCD) registry to (i) quantify age-adjusted mortality from appendiceal disease (International Classification of Diseases, Tenth Revision (ICD-10) K35-K38) with septicemia (ICD-10 A41) as a contributing cause among U.S. adults aged ≥25 years over 1999 to 2020, (ii) identify demographic subgroups with the highest age-adjusted mortality burden, and (iii) characterize temporal trends in age-adjusted mortality across the study period, including changes that may correspond to recent disruptions in surgical and emergency care. As a descriptive epidemiologic analysis based on death certificate data, the study is intended to characterize mortality patterns rather than establish causal mechanisms.

## Materials and methods

Data source and ethical considerations

We performed a retrospective, population-based analysis using publicly available, de-identified death certificate records from the CDC WONDER MCD database [7], covering all deaths registered in the United States between 1999 and 2020. Because the dataset consists of publicly available, de-identified mortality records, the study did not require Institutional Review Board approval under 45 CFR 46.102(e)(1) [8]. Data were extracted from the CDC WONDER online query interface on August 18, 2025.

Case selection and query parameters

We identified all deaths in adults aged ≥25 years for which appendiceal disease (ICD-10 codes K35-K38) was assigned as the underlying cause of death and septicemia (ICD-10 codes A41.0-A41.9) appeared among the multiple causes of death. The age threshold of ≥25 years was chosen to exclude pediatric and young-adult cases. The ≥25 threshold was selected to focus on mature adult mortality patterns and reduce heterogeneity introduced by pediatric and young-adult presentations. We chose this specific combination of codes to characterize the population-level mortality burden associated with septicemia as a contributing complication, given prior evidence that appendectomy is associated with a 1.29-fold increased risk of subsequent sepsis [9,10].

Within the CDC WONDER interface, the following parameters were applied: underlying cause of death restricted to K35-K38; multiple cause of death restricted to A41.0-A41.9; 10-year age groups limited to 25-34, 35-44, 45-54, 55-64, 65-74, 75-84, and 85+ years; all U.S. states and the District of Columbia included; all sexes, races, and ethnicities included; calendar years 1999 through 2020. Mortality counts and rates were extracted by sex, race (American Indian or Alaska Native, Asian or Pacific Islander, Black or African American, White), 2013 National Center for Health Statistics (NCHS) Urban-Rural Classification Scheme for Counties (Large Central Metro, Large Fringe Metro, Medium Metro, Small Metro, Micropolitan, NonCore), place of death, and year of death. The overall analysis retained cases with missing demographic information; stratified analyses excluded such cases for the variable under consideration. To provide context for the magnitude of the burden attributable to septicemia, a parallel query without the A41 multiple-cause filter was extracted to obtain total appendiceal disease mortality for the same population and period.

Statistical analysis

Crude and age-adjusted mortality rates (AAMRs) per 1,000,000 population were calculated within the CDC WONDER system, with 95% confidence intervals (CIs) generated by CDC using the gamma distribution method for counts under 100 and the Tiwari modification of the standard error method for counts of 100 or more. Age standardization was performed against the 2000 U.S. standard population using the 10-year age groups specified above. Mortality counts below 10 within any stratum were suppressed in the CDC output in accordance with CDC WONDER confidentiality policy; corresponding rates were either suppressed or flagged as unreliable and were excluded from quantitative trend estimation in the affected stratum-years.

Temporal trends in AAMR were analyzed using joinpoint regression with Joinpoint Trend Analysis Software, version 5.3 (Statistical Methodology and Applications Branch, Surveillance Research Program, National Cancer Institute; November 2024 release). The model was configured with the grid-search method, a minimum of zero and a maximum of three joinpoints per series, a minimum of two observations from a joinpoint to either end of the series, and a minimum of two observations between adjacent joinpoints. Model selection was based on the permutation test with an overall significance level of 0.05. Annual percent changes (APCs) and their 95% CIs were estimated for each identified trend segment, with APCs whose p-value was below 0.05 considered statistically significantly different from zero. Joinpoint analyses were performed for the overall cohort and stratified by sex and race; race-stratified joinpoint analysis was attempted for all available racial categories but was feasible only for the White population because annual death counts fell below 10 in multiple years for the remaining groups, precluding stable trend estimation. Place of death was reported only as counts and percentages because place of death is an attribute of the death event and has no corresponding population denominator.

## Results

Eligible cohort

Across the United States from 1999 to 2020, a total of 3,923 deaths in adults aged ≥25 years met inclusion criteria, in which appendiceal disease (ICD-10 K35-K38) was assigned as the underlying cause of death and septicemia (ICD-10 A41.0-A41.9) appeared among the multiple causes of death. The crude mortality rate for this combination was 0.9 per million population (95% CI 0.8-0.9), with an AAMR of 0.8 per million (95% CI 0.8-0.9).

Demographic distributions and corresponding mortality rates are presented in Table 1 and Figure 2. Mortality was higher among males than females in both crude and age-adjusted estimates: males accounted for 2,193 deaths (55.9%) with an AAMR of 1.1 per million (95% CI 1.0-1.1), compared with 1,730 deaths (44.1%) and an AAMR of 0.6 per million (95% CI 0.6-0.7) among females. The age-adjusted male-to-female mortality ratio was approximately 1.8, indicating a substantial sex disparity that persisted after accounting for differences in age structure.

**Table 1:** Demographic and clinical characteristics of appendiceal disease deaths with septicemia as a contributing cause, United States, 1999 to 2020 *Crude rate per 1,000,000 population. †AAMR: Age-Adjusted Mortality Rate per 1,000,000, standardized to the 2000 U.S. standard population. Underlying cause of death: Diseases of appendix (ICD-10 K35–K38); Multiple cause of death: Sepsis (ICD-10 A41.0–A41.9). Adults aged ≥25 years. Rates not reported for "Place of death" stratification per CDC WONDER (place of death is an attribute of the death event and has no population denominator). "Suppressed" indicates counts <10 per CDC confidentiality policy. Source: CDC WONDER, Multiple Cause of Death, 1999–2020 [7].

Characteristic	Deaths (n)	% of Total	Crude Rate* (95% CI)	AAMR† (95% CI)
Overall (1999-2020)	3923	1	0.9 (0.8-0.9)	0.8 (0.8-0.9)
Sex				
Male	2193	0.559	1.0 (1.0-1.1)	1.1 (1.0-1.1)
Female	1730	0.441	0.7 (0.7-0.8)	0.6 (0.6-0.7)
Race				
American Indian or Alaska Native	40	0.01	0.8 (0.6-1.1)	1.1 (0.7-1.5)
Black or African American	503	0.128	0.9 (0.8-1.0)	1.0 (1.0-1.1)
White	3270	0.834	0.9 (0.9-0.9)	0.8 (0.8-0.8)
Asian or Pacific Islander	110	0.028	0.4 (0.4-0.5)	0.6 (0.5-0.7)
Urbanization (2013 NCHS classification)				
Large Central Metro	1115	0.284	0.8 (0.8-0.9)	0.9 (0.8-0.9)
Large Fringe Metro	799	0.204	0.7 (0.7-0.8)	0.7 (0.7-0.8)
Medium Metro	854	0.218	0.9 (0.9-1.0)	0.9 (0.8-0.9)
Small Metro	390	0.099	1.0 (0.9-1.1)	0.9 (0.8-0.9)
Micropolitan (Nonmetro)	439	0.112	1.1 (1.0-1.2)	0.9 (0.9-1.0)
NonCore (Nonmetro)	326	0.083	1.1 (1.0-1.3)	0.9 (0.8-1.0)
Place of death				
Medical Facility - Inpatient	3308	0.843	-	-
Decedent’s home	182	0.046	-	-
Nursing home / long-term care	124	0.032	-	-
Hospice facility	121	0.031	-	-
Medical Facility - Outpatient or ER	113	0.029	-	-
Other	53	0.014	-	-
Place of death unknown	16	0.004	-	-
Medical Facility - Dead on Arrival	Suppressed	-	-	-
Medical Facility - Status unknown	Suppressed	-	-	-

**Figure 1:**
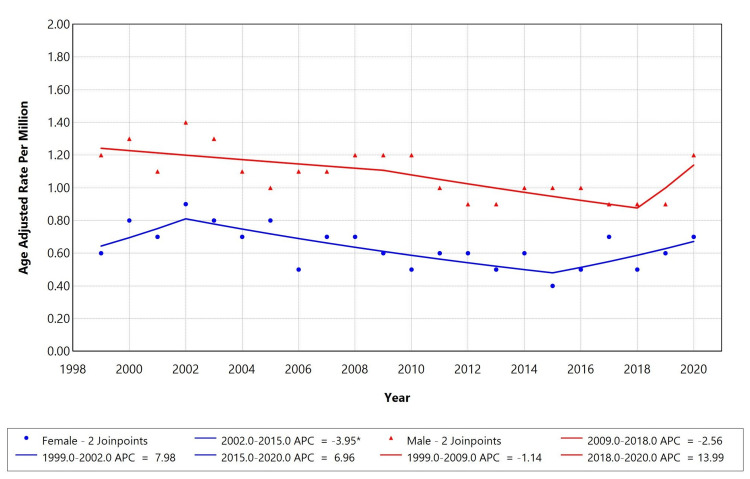
Sex stratified age-adjusted mortality patterns in U.S. adults aged 25 and above, 1999 to 2020. APC = Annual Percent Change

Although White individuals accounted for the largest absolute number of deaths (3,270; 83.4%), AAMRs revealed a different pattern of risk. The highest AAMR was observed among American Indian or Alaska Native persons (1.1 per million; 95% CI 0.7-1.5), followed by Black or African American persons (1.0 per million; 95% CI 1.0-1.1), White persons (0.8 per million; 95% CI 0.8-0.8), and Asian or Pacific Islander persons (0.6 per million; 95% CI 0.5-0.7). The wide confidence interval for the American Indian/Alaska Native estimate reflects the small absolute count (n = 40) and should be interpreted with caution. These rate-based comparisons indicate that the burden of fatal appendiceal disease with septicemia is not greatest among White individuals when expressed on a per-population basis, despite the predominance of this group in absolute counts.

When stratified by the 2013 NCHS urbanization classification, crude mortality rates were modestly higher in nonmetropolitan than metropolitan settings (1.1 per million in both Micropolitan and NonCore Nonmetro categories versus 0.7-1.0 across metropolitan categories). However, this gradient largely disappeared after age standardization: AAMRs were 0.7-0.9 per million across all six urbanization categories, with overlapping confidence intervals. This pattern indicates that the apparent rural excess in crude rates is largely attributable to the older age structure of nonmetropolitan populations rather than to a true elevation in age-specific mortality risk.

The majority of deaths occurred in inpatient medical facilities (n = 3,308; 84.3%); when outpatient and emergency department deaths were included, all medical facility settings together accounted for 87.2% of deaths (n = 3,421). Deaths occurring at the decedent’s home (4.6%), in nursing homes or long-term care facilities (3.2%), and in hospice facilities (3.1%) made up most of the remaining mortality. Two place-of-death categories (Dead on Arrival, Status unknown) had counts <10 and were suppressed per CDC confidentiality policy. Mortality rates are not reported for the place-of-death stratification because place of death is an attribute of the death event rather than of a living population at risk and therefore has no valid denominator.

**Figure 2:**
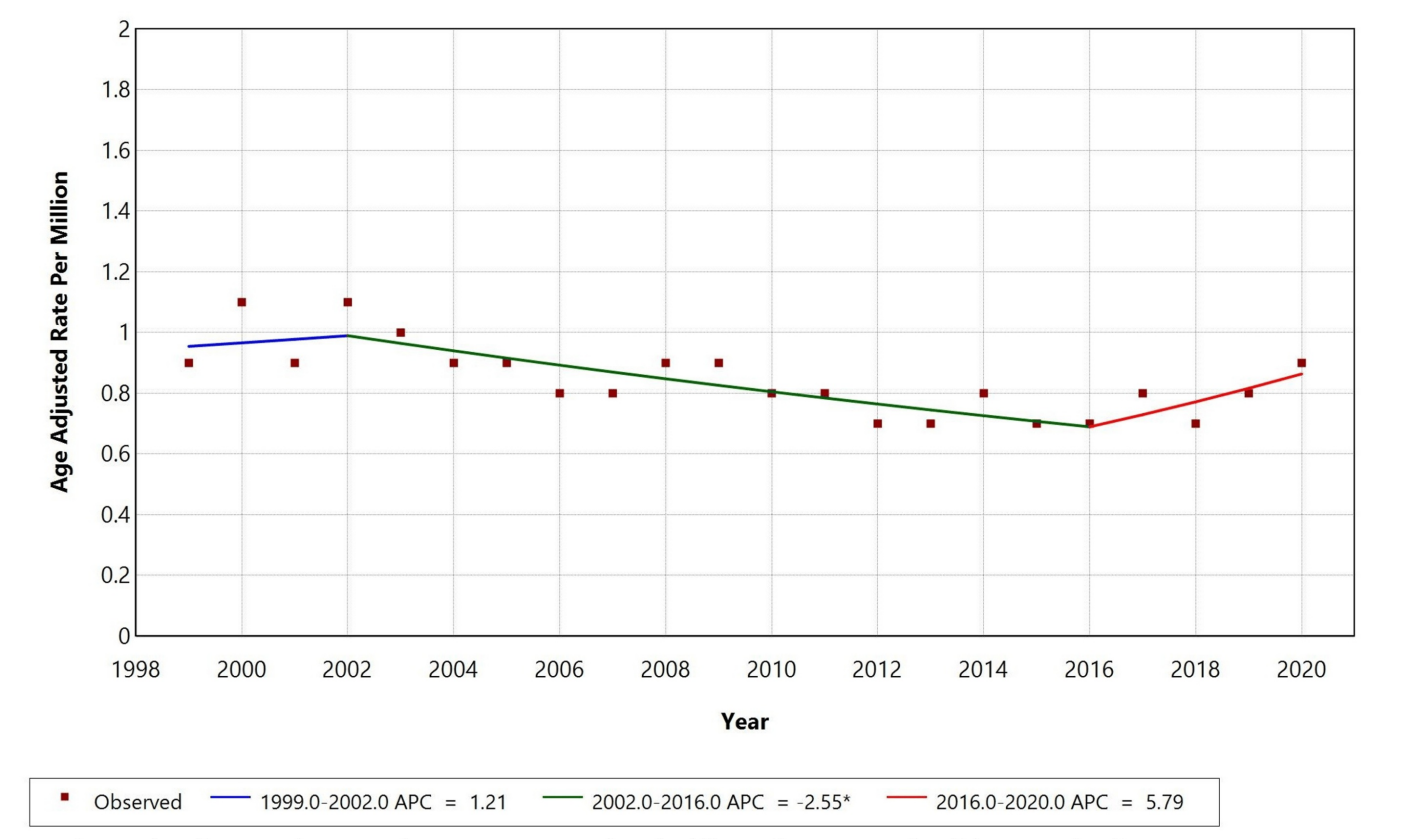
Age-adjusted mortality trends with joinpoint analysis for White individuals aged 25 and above, United States, 1999 to 2020. APC = Annual Percent Change

Joinpoint regression of overall age-adjusted mortality identified three distinct trend segments across 1999 to 2020 (Table 2, Figure 2). AAMR increased between 1999 and 2002 (APC +1.21%), although this trend was not statistically significant (p = 0.16). Mortality then declined significantly between 2002 and 2016 (APC −2.55%; p < 0.001), followed by a significant increase between 2016 and 2020 (APC +5.79%; p = 0.02). The most recent segment overlaps with the COVID-19 pandemic period and should be interpreted with caution, as pandemic-related disruptions to surgical care, emergency department access, and death certification practices may have contributed to the observed reversal independently of underlying disease trends.

**Table 2:** Joinpoint regression of age-adjusted mortality trends for appendiceal disease with septicemia as a contributing cause, U.S. adults aged ≥25 years, 1999 to 2020 APC: Annual Percent Change. CI: Confidence Interval. *Statistically significant (p < 0.05): the trend is significantly different from zero. ‡Final trend segment overlaps with the COVID-19 pandemic period (2020); estimates for this segment should be interpreted with caution given pandemic-related disruptions to surgical care, healthcare access, and death certification practices. §Joinpoint trend analysis was not feasible for Black or African American, Asian or Pacific Islander, and American Indian or Alaska Native populations because annual death counts were below 10 in multiple years and were suppressed per CDC confidentiality policy. Stable trend estimates could not be generated for these subgroups. Joinpoint regression performed using Joinpoint Trend Analysis Software, version 5.3 (November 2024), National Cancer Institute.

Stratum	Trend Segment	APC (95% CI)	p-value	Direction
Overall (U.S. adults ≥25 years)				
Overall	1999-2002	+1.21 (−0.5, +2.9)	0.16	Increasing
Overall	2002-2016	−2.55 (−3.4, −1.7)	<0.001	Decreasing*
Overall	2016-2020‡	+5.79 (+1.2, +10.6)	0.02	Increasing*
Sex-stratified				
Male	1999-2009	−1.14 (−2.4, +0.1)	0.08	Decreasing
Male	2009-2018	−2.56 (−4.1, −1.0)	0.003	Decreasing*
Male	2018-2020‡	+13.99 (+1.5, +28.0)	0.03	Increasing*
Female	1999-2002	+7.98 (−4.5, +22.1)	0.21	Increasing
Female	2002-2015	−3.95 (−5.4, −2.5)	<0.001	Decreasing*
Female	2015-2020‡	+6.96 (−0.4, +14.9)	0.06	Increasing
Race-stratified				
White	1999-2002	+2.00 (−1.8, +5.9)	0.3	Increasing
White	2002-2018	−1.15 (−2.0, −0.3)	0.01	Decreasing*
White	2018-2020‡	+11.24 (−0.5, +24.4)	0.06	Increasing
Black or African American	-	Not estimated§	-	-
Asian or Pacific Islander	-	Not estimated§	-	-
American Indian or Alaska Native	-	Not estimated§	-	-

Among males, AAMR showed a non-significant decline between 1999 and 2009 (APC -1.14%; p = 0.08), followed by a significant decline between 2009 and 2018 (APC -2.56%; p = 0.003). Mortality then increased significantly between 2018 and 2020 (APC +13.99%; p = 0.03). Among females, AAMR increased between 1999 and 2002 (APC +7.98%), although this trend was not statistically significant (p = 0.21). Mortality then declined significantly between 2002 and 2015 (APC −3.95%; p < 0.001), followed by a non-significant increase between 2015 and 2020 (APC +6.96%; p = 0.06).

**Figure 3:**
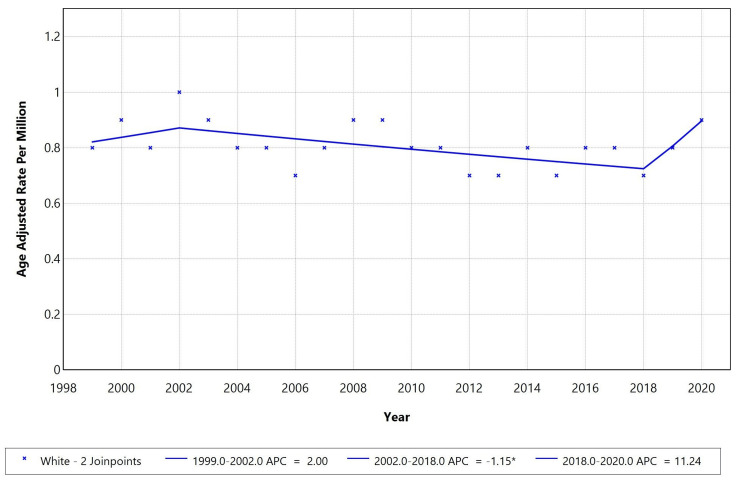
Age-adjusted mortality patterns by sex among adults aged 25 and above in the United States, 1999 to 2020 APC = Annual Percent Change

Race-stratified joinpoint analysis (Figure 3) was feasible only for the White population. Among White individuals, AAMR increased between 1999 and 2002 (APC +2.00%), although this trend was not statistically significant (p = 0.30). Mortality then declined significantly between 2002 and 2018 (APC -1.15%; p = 0.01), followed by a non-significant increase between 2018 and 2020 (APC +11.24%; p = 0.06). Joinpoint regression could not be performed for Black or African American, Asian or Pacific Islander, or American Indian or Alaska Native populations because annual death counts were below 10 in multiple years across the study period and were therefore suppressed under the CDC WONDER confidentiality policy. Stable trend estimates were not generated for these subgroups, and no inference regarding temporal change in mortality among populations other than White can be drawn from the present analysis.

To contextualize the population-level burden of septicemia-related appendiceal mortality, we compared the study cohort against all appendiceal disease deaths in U.S. adults ≥25 years during the same period (Table 3). A total of 8,549 deaths were attributed to appendiceal disease (K35-K38) as the underlying cause, corresponding to a crude mortality rate of 1.9 per million (95% CI 1.9-2.0) and an AAMR of 1.8 per million (95% CI 1.8-1.9). The 3,923 deaths in which septicemia was also recorded represented 45.9% of all appendiceal disease deaths, indicating that septicemia is implicated in nearly half of all fatal appendiceal disease in this population and is therefore a dominant pathway to death rather than a rare complication.

**Table 3:** Mortality from appendiceal disease overall and with septicemia as a contributing cause, U.S. adults aged ≥25 years, 1999 to 2020 *Crude rate per 1,000,000 population. †AAMR: Age-Adjusted Mortality Rate per 1,000,000, standardized to the 2000 U.S. standard population. Population denominator: 4,473,854,489 person-years (U.S. adults aged ≥25 years, 1999–2020). Septicemia was listed as a contributing cause of death in 45.9% of all deaths in which appendiceal disease was the underlying cause. Source: CDC WONDER, Multiple Cause of Death, 1999–2020 [7]

Cohort	Deaths (n)	Crude Rate* (95% CI)	AAMR† (95% CI)	% of all appendiceal deaths
All appendiceal disease (ICD-10 K35–K38)	8549	1.9 (1.9–2.0)	1.8 (1.8–1.9)	1
With septicemia contributing (ICD-10 A41.0–A41.9)	3923	0.9 (0.8–0.9)	0.8 (0.8–0.9)	0.459

## Discussion

This retrospective analysis of the CDC WONDER Multiple Cause of Death registry examined mortality from appendiceal disease (ICD-10 K35-K38) with septicemia (ICD-10 A41) as a contributing cause in U.S. adults aged ≥25 years between 1999 and 2020. A total of 3,923 such deaths were identified, representing 45.9% of all appendiceal disease deaths in this population over the same period and underscoring that septicemia is not a rare complication but a dominant pathway to death in fatal appendiceal disease. AAMRs revealed important demographic patterns that differ from those suggested by absolute counts alone. Although White individuals accounted for the largest absolute number of deaths (83.4%), age-adjusted mortality was highest among American Indian or Alaska Native and Black or African American populations, indicating that the per-population burden of fatal disease is disproportionately borne by these groups despite their smaller representation in absolute counts. Mortality was also higher in men than women on an age-adjusted basis (AAMR 1.1 vs 0.6 per million), consistent with prior reports of greater appendicitis and sepsis incidence in males. The overwhelming majority of deaths (84.3%) occurred in inpatient medical facilities, indicating that most fatal cases reach hospital care but do not survive admission.

The progression from appendiceal disease to septicemia involves complex pro-inflammatory pathways that culminate in systemic inflammatory response and organ dysfunction [5]. Septicemia is well recognized as a life-threatening, dysregulated host response to infection [3], and the mortality risk rises sharply when sepsis is complicated by cardiac arrest, acute renal failure, or major bleeding [11]. Bloodstream infections in the setting of acute appendicitis are independently associated with worsened postoperative outcomes regardless of co-existing complications [12]. While our descriptive design cannot directly demonstrate the impact of any specific clinical intervention, these established pathophysiological links provide context for why septicemia features so prominently in fatal appendiceal disease at the population level.

Hospital-based studies of appendicitis mortality consistently report higher case-fatality rates than those derived from population-level data, and the two are not directly comparable because hospital cohorts capture only patients reaching care. Potey et al. reported 4.8% in-hospital mortality for perforated appendicitis, approximately half of which was attributable to septicemia [12]. Williams et al. observed mortality of 5.6% in resource-limited settings compared with 0.24% in developed countries [13], illustrating how access to surgical and intensive care shapes survival. Wu et al. reported a postoperative sepsis incidence of 0.43% and a 30-day mortality of 5.47% among septic patients [9]. Population-based rates, such as the AAMR of 0.8 per million observed in our study, capture the residual mortality burden that remains after the contemporary U.S. healthcare system’s efforts to prevent, detect, and treat appendicitis.

Geographic and demographic patterns require careful interpretation. The absolute concentration of deaths in metropolitan areas (80.5%) reflects population distribution rather than excess per-capita risk: when age-adjusted, mortality rates were essentially flat across all six NCHS urbanization categories (AAMR 0.7-0.9 per million), and the modest crude-rate elevation in nonmetropolitan areas (1.1 per million) was largely explained by the older age structure of those populations. The male predominance in deaths (55.9%) corresponds to a meaningful age-adjusted disparity (AAMR ratio approximately 1.8), aligning with prior reports of higher appendicitis and sepsis incidence in men. Racial patterns, however, warrant a substantially different interpretation than absolute counts would suggest. Although White individuals account for the largest share of deaths in absolute terms, the highest AAMRs were observed among American Indian or Alaska Native and Black or African American populations. Several mechanisms may contribute to such disparities, including differential access to emergency surgical care, delays in presentation, and a higher prevalence of comorbid conditions that worsen sepsis outcomes [14]; African American race has also been identified as a risk factor for post-appendectomy sepsis in prior work [4]. Our descriptive data cannot directly test any of these mechanisms but support the need for further investigation into the drivers of these inequities.

The finding that 84.3% of deaths occurred in inpatient facilities, with an additional 2.9% in outpatient or emergency department settings, indicates that most fatal appendiceal disease with septicemia occurs after the patient has reached hospital care rather than before [15]. This pattern is consistent with the natural history of complicated appendicitis, in which deterioration to severe sepsis typically follows hospital presentation. Deaths at the decedent’s home (4.6%), in nursing homes (3.2%), and in hospice facilities (3.1%) likely represent a heterogeneous group that may include patients with rapid clinical decline, those with limited access to timely emergency care, and patients with significant pre-existing illness for whom aggressive intervention was not pursued.

Temporal analysis revealed a statistically significant decline in age-adjusted mortality between 2002 and 2016 (APC -2.55%), suggesting meaningful population-level progress in the management of complicated appendicitis during this period. This contrasts with the relatively stable crude mortality rate of 0.9 per million across the full study window, which masks the underlying age-adjusted improvement because the U.S. population grew older over the same period. The renewed increase observed after 2016, and particularly during 2018-2020 across multiple strata, coincides with the COVID-19 pandemic, which is known to have disrupted access to emergency surgical care, delayed appendectomies, and altered death certification practices. Whether this terminal rise reflects a true reversal of prior gains, pandemic-related disruptions, or both cannot be determined from our data, and the brevity of the affected segments (two to three years) limits the precision of these estimates. Sustained surveillance beyond 2020 will be necessary to clarify whether the post-2016 increase persists or returns to the prior declining trajectory. Surgical delays exceeding 24 hours have been associated with an 81% increase in postoperative mortality [9], supporting the hypothesis that pandemic-related delays could have contributed to the observed uptick.

Advanced age, comorbidities, and delayed presentation are established risk factors for mortality in complicated appendicitis. Early surgical intervention can limit mortality to below 1% even with a 51% complication rate, and bloodstream infections in appendicitis approximately double postoperative morbidity regardless of surgical approach [12]. Although our descriptive analysis cannot directly evaluate the effect of specific interventions, the findings are consistent with established clinical evidence supporting strategies such as public education on early symptom recognition, rapid triage of suspected appendicitis, and standardized sepsis management pathways in emergency settings. Identifying which patient groups bear the highest age-adjusted mortality burden may help target such interventions where they are most likely to reduce inequities in outcome.

Limitations

As an analysis of death certificate data, this study is subject to coding inaccuracies and variability in how septicemia is recorded across jurisdictions and over time; septicemia is also frequently over-reported as a terminal event, which may inflate its apparent contribution. Evolving diagnostic and reporting practices over the 22-year period may have influenced observed trends. The Multiple Cause of Death dataset lacks information on comorbidities, time to surgery, hospital characteristics, and socioeconomic status, so this study can describe demographic patterns but cannot identify their causal drivers. Because only death counts are available, we cannot distinguish whether elevated mortality in particular groups reflects higher disease incidence, higher case-fatality, or both. For racial subgroups other than White, annual counts below 10 were suppressed in multiple years, precluding joinpoint trend estimation. As a descriptive analysis, the study generates hypotheses but cannot establish causation, and geographic findings reflect population-level rather than individual-level associations.

## Conclusions

This nationwide analysis demonstrates that septicemia is implicated in nearly half (45.9%) of all deaths from appendiceal disease in U.S. adults aged ≥25 years and is therefore a dominant pathway to fatal outcome rather than a rare complication. Age-adjusted mortality declined significantly between 2002 and 2016, reflecting meaningful population-level progress, but rose again after 2016, with the terminal segment overlapping the COVID-19 pandemic period. Mortality burden was not uniform across demographic groups: age-adjusted rates were highest among American Indian or Alaska Native and Black or African American populations and among men, indicating that the per-population burden of fatal disease is disproportionately borne by these groups despite White individuals accounting for the largest share of absolute deaths. The overwhelming majority of deaths occurred in inpatient settings, indicating that most fatal cases reach hospital care but do not survive admission. These findings highlight the need for sustained surveillance to clarify whether the post-2016 increase represents a persistent reversal or a pandemic-related disruption, and for further research into the clinical and structural factors that drive the observed demographic inequities. Although this descriptive analysis cannot identify causal mechanisms or evaluate specific interventions, the findings are consistent with established clinical evidence supporting timely recognition of appendicitis, prompt surgical management, and standardized sepsis care, particularly in groups and settings where the age-adjusted mortality burden is greatest.
